# Identifying targets for interventions to improve communication between primary and secondary care: a qualitative study of referrals to adult NHS hearing aid services

**DOI:** 10.1186/s13690-025-01756-4

**Published:** 2025-11-26

**Authors:** Sobia Khan, Michael T. Loughran, Christopher J. Armitage, Ted Leverton, Nishchay Mehta, Francesca Oliver, Martin O’Driscoll, Greg Nassar, Chris J. Sutton, Kathryn Lewis, Piers Dawes, Peter Bower, Kevin J. Munro

**Affiliations:** 1 Manchester Centre for Audiology and Deafness, School of Health Sciences, University of Manchester, Manchester, UK; 2 Manchester Centre for Health Psychology, Division of Psychology and Mental Health, The University of Manchester, Manchester Academic Health Science Centre, Manchester, UK; 3 NIHR Greater Manchester Patient Safety Research Collaboration, The University of Manchester, Manchester Academic Health Science Centre, Manchester, UK; 4 Centre for Hearing Research, School of Health and Rehabilitation Sciences, The University of Queensland, Brisbane, Australia; 5 Centre for Primary Care and Health Services Research, Faculty of Biology, Medicine and Health, The University of Manchester, Manchester, UK; 6 Manchester Academic Health Science Centre, Manchester University Hospitals NHS Foundation Trust, Manchester, UK; 7 Retired GP and Clinical Advisor for Royal College of General Practitioners, London, England; 8 National Institute for Health Care Research, University College London Hospital Biomedical Research Centre Hearing Health Theme, London, UK; 9 Royal National Ear, Nose, and Throat & Eastman Dental Hospital, University College London Hospitals NHS Foundation Trust, London, UK; 10 The Royal National Institute for Deaf People, Peterborough, UK; 11 Manchester Royal Infirmary and Royal Manchester Children’s Hospital, Manchester University Hospitals NHS Foundation Trust, Manchester, UK; 12 Trafford General Hospital, Manchester University Hospitals NHS Foundation Trust, Manchester, UK; 13 Lancashire Clinical Trials Unit, Applied Health Research Hub, University of Lancashire, Preston, UK; 14 Withington Community Hospital and Wythenshawe Hospital, Manchester University Hospitals NHS Foundation Trust, Manchester, UK

**Keywords:** Primary care, Secondary care, General practice, Adult hearing aid services, Audiology, Communication, Collaboration, Behaviour change, Organisational change

## Abstract

**Background:**

Improving communication between NHS primary and secondary care services should reduce inefficiencies, improve patient care, and provide a source of accurate data for audit and research. Using general practice (primary care) and adult hearing aid services (secondary care) as examples, this study aimed to investigate the: (i) information primary care want from secondary care services during referral processes and what information secondary care can provide, (ii) barriers and facilitators for coding and providing this information, and (iii) targets for interventions to improve communication.

**Methods:**

Qualitative interview study. Twenty-nine semi-structured interviews were conducted with primary and secondary care staff in North West England, informed by the capabilities, opportunities, and motivations model of behaviour change (COM-B). A thematic analysis with findings mapped to the Theoretical Domains Framework (TDF) was used to identify barriers and facilitators to the recording and communication of hearing health information, as well as behavioural determinants to target in future interventions.

**Results:**

Four key TDF domains were identified as potential determinants to target for future interventions: (1) time and resources (e.g., environmental context and resources) are stretched across the two sectors; (2) fatigue, tiredness, and cognitive overload affecting workflow (e.g., memory, attention, and decision processes), particularly adult hearing aid service administration; (3) adult hearing aid services are unaware if their letters are read by general practice, or if the level of information they send is appropriate (e.g., knowledge); and (4) difficulties communicating with general practice and a lack of feedback is causing reduced motivation from adult hearing aid services to make direct contact (e.g., beliefs about consequences).

**Conclusions:**

This study identified four theoretically-derived variables that are barriers to communication and record sharing, and highlights how these can be translated into potential interventions (e.g., simplified coding). Further work is required to test interventions developed using behaviour change theory.

**Supplementary Information:**

The online version contains supplementary material available at 10.1186/s13690-025-01756-4.

**Table:** 

Text box 1. Contributions to the literature	
•This study is unique in that it is able to compare and contrast within a single paper the viewpoints of primary and secondary care services staff that routinely interact with one another. •At a time when NHS services are under pressure to deliver a better working collaboration between primary and secondary care to help improve patient care and interaction with one another, this paper provides theoretically-derived variables that can be applied to interventions to help overcome some of these challenges. •Data generated from this paper has been applied to a preliminary study and it has shown promising results.	

## Background

The NHS Confederation, a body to support and represent providers of UK NHS services, has called for a stronger working collaboration between primary and secondary care services in order to deliver the best care to patients [1]. Primary care is the first point of contact and secondary care is where referrals are made to specialist services. Collaboration between primary and secondary care can lead to benefits for both sectors (e.g., improved relationships), including patient confidence in services [1], and accurate data sources for audit and research purposes [2, 3]. Healthcare professionals have suggested key areas to focus efforts for stronger collaborations between primary and secondary care (e.g., sharing of information) [1].

Previous research investigating communication and collaboration between primary care and a broad range of secondary care services found that system changes may be required [4], that there is a need for more open lines of communication and improved record sharing [5], that collaboration can be a complex process [6], and an overarching need for robust interventions to improve communication and collaboration [4–6].

General practice and adult hearing aid services (i.e. audiology) represent two separate services provided by the NHS but usually independently and in different locations. Information on the provision of hearing aids is currently recorded by both services; however, this information is saved in a variety of IT systems, with adult hearing aid services having their own patient management systems (i.e. Auditbase, or Practice Navigator) that are not linked to electronic medical records used by general practice (Egton Medical Information Services [EMIS], Docman). Variations in how the data are handled and shared can impact on the utility of the information for both clinical and research purposes. Currently, it is not possible to track the provision of hearing aids in primary care because of the variations in how information is communicated and recorded. Within the NHS there is a need to communicate and record data accurately [2] to drive discovery, identify and address health care needs, and assist robust responses to government and policymakers [7]. Little is known about the differences or similarities of how these two groups of professionals communicate and record referral information between one another, nor do we know about their drivers of behaviour.

In alignment with the NHS Confederation call for improvements, for the first time, the present qualitative research examined, in depth, communication and the recording of information during referral processes between both general practice and adult hearing aid services (i.e. referral to secondary care for hearing assessment within adult audiology, who can treat the patient and relay information back to primary care), and how this informs what future interventions might look like to improve collaboration. Improvements in collaboration between the two sectors could lead to national audit and research opportunities similar to the National Respiratory Audit Programme (NRAP) by the Royal College of Physicians, which utilises primary care data [3].

Hearing loss is ranked third in total years lived with a disability [8], causing communication issues, reduced quality of life (QoL), and associations with anxiety, depression and dementia [9–11]. Hearing aids can help overcome some of these challenges [12, 13]; however, although there has been a great deal of research on hearing aid uptake in terms of patient behaviour and influencing factors [14–16], research shows that uptake of hearing aids is both low and slow [17–19]. As a result, there have been frequent calls for a national adult hearing screening programme and attempts to raise awareness (e.g., online hearing screening from World Health Organization and Royal National Institute for Deaf People [20, 21].

The NHS hearing pathway accounts for 80% of hearing aids distributed in the UK per year [22] at a cost of £450 million to the NHS [23]. It is not only NHS secondary care adult hearing aid services that supply these, independent providers of health care are sometimes commissioned on behalf of the NHS, including some high street hearing aid dispensers (e.g., Specsavers). However, for the purposes of this study, only secondary care services are considered. The pathway itself can be broken down into seven stages of hearing health seeking [17, 18]:

1. Did not report difficulties.
2. Reported difficulties
3. Told a healthcare professional about difficulties
4. Referred for an assessment
5. Offered intervention
6. Accepted intervention
7. Reported compliance with intervention

With such a large population in need of adult hearing aid services and large sums of money involved, each step represents a potential opportunity for improved efficiency and cost-effectiveness for the NHS. Sawyer et al. [18] suggested that interventions for each stage of the pathway need to be examined separately because they have different drivers of behaviour. For example, if we look at the top and tail of the pathway, we know that it takes patients approximately nine years to seek help from when they first recognise hearing difficulties [24], and of those that are offered and accept hearing aids, 20% are not used at all [25]. Historically it has been reported that general practitioners were a barrier to hearing aid use by not referring people for a hearing assessment [26, 27]; however, Sawyer et al. [18] reported that only 4.5% of people who reported hearing difficulties did not get offered a referral for a hearing assessment. Beyond this, relatively little is known about stage four of the pathway from the point that a referral is opened (i.e. primary care refers patients to adult hearing aid services) until it is closed (i.e. audiology services inform primary care of outcome of original referral). Currently, we don’t know the how and why data is communicated, how relevant is the information, and what is driving the behaviour for both sectors. Delving deeper into the communication between primary and secondary care during the referral for a hearing assessment stage of the pathway represents an opportunity to reduce inefficiencies, improve patient care, provide a source of accurate data for audit and research, and improve future collaboration.

The present research was guided by the Behaviour Change Wheel (BCW) [28, 29], a framework synthesised from 19 other frameworks to aid systematic intervention development. The BCW is grounded in behaviour change theory and satisfies the MRC outline for theory-based approaches to intervention design [30]. To the best of our knowledge, the BCW has not been utilised as a theoretical framework to assess the recording and communication of hearing health care information between general practice and adult hearing aid services and to inform future interventions. Thus, for the first time, we utilise the theoretical constructs of the BCW to enable exploration of, and development of, potential barriers and facilitators of behaviour change for both care sectors to be incorporated into intervention design, from the point that stage four of the hearing health seeking pathway begins and ends.

Using general practice and adult hearing aid services as examples, the aims of this study were to investigate the [1] information primary care want from secondary care services during referral processes and what information secondary care can provide [2], barriers and facilitators there are for coding and providing this information, and [3] identify behavioural determinants for future interventions to target.

## Methods

### Setting and participants

Ethical approval was granted through Integrated Research Application System (IRAS) (REF: 295139/1545834/37/936). Semi-structured interviews were conducted across NHS general practice (n=15) and adult hearing aid service (n=14) staff members at sites in the North West of England to gather qualitative data through purposive sampling, thus enabling a range of views to be gathered from both perspectives. The careers of participants across both sectors varied from those at the start of their careers to others with more than 25 years of experience; adult hearing aid service staff had an even mix of audiologists and administration staff; general practice staff were predominantly administrative, with a small number of general practitioners. The participants were aged between 18 and 67 years, with seven males and 22 females. Seven participants were Non-White, and 22 were White-British. The number of people approached who chose not to participate, and the reasons for their non-participation, are not known.

Interviews were conducted in the workplace, with no-one else present in the room allowing participants to speak freely. All interviews were carried out by female researcher SK, with training and guidance on how to conduct qualitative interviews provided by CJA, whom has a high level of experience within qualitative behaviour change research. Apart from building a friendly rapport with lead members of each site in order to be able to conduct interviews, SK has no professional or research connections within general practice or audiology services, and participants had no prior knowledge of SK’s background other than standard university credentials, therefore removing any bias during interviews. Data saturation was perceived to have been attained by the 29th interview as no new data was being attained that provided novel findings; this is appropriate according to criteria outlined by Braun & Clarke [31].

### Procedure

Each service was contacted by telephone or email to identify names of relevant staff that would be willing to participate, with further contact details then supplied by each site. A reminder email was sent after one week from the initial contact.

Participants were provided with a detailed information sheet, and had the opportunity to ask questions and discuss the project with the researchers. Written consent was obtained when interviews were conducted face to face. Verbal consent was obtained when using video conferencing by asking the participant a series of questions (through the use of a consent script) and recording their verbal agreement to each statement. Twenty-eight interviews were conducted face-to-face and one conducted via Zoom. Staff were reimbursed in recognition of their time for participation. Interviews lasted for approximately 20 min. Transcripts were not returned to participants post-interview, and participants were not asked for feedback on final findings. Interviews were audio recorded via encrypted devices, with all identifiable data removed from transcripts before analysis and stored securely at the University of Manchester.

### Design

General practice staff were asked if they felt they received sufficient information from adult hearing aid services to do their jobs accordingly, and what further information they felt would be helpful. This helped assess if staff currently had enough information to enact ‘behaviour’ (i.e. process incoming documentation from adult hearing aid services with regards the outcome of new adult hearing aid referrals).

‘Behaviour’ (i.e. sending documentation to general practice highlighting the outcome of the initial referral for hearing assessment) of adult hearing aid service staff was assessed by asking questions about the current information they were sending to primary care, if they were ever contacted to send further information, and would they be willing to send more information.

**Fig. 1:**
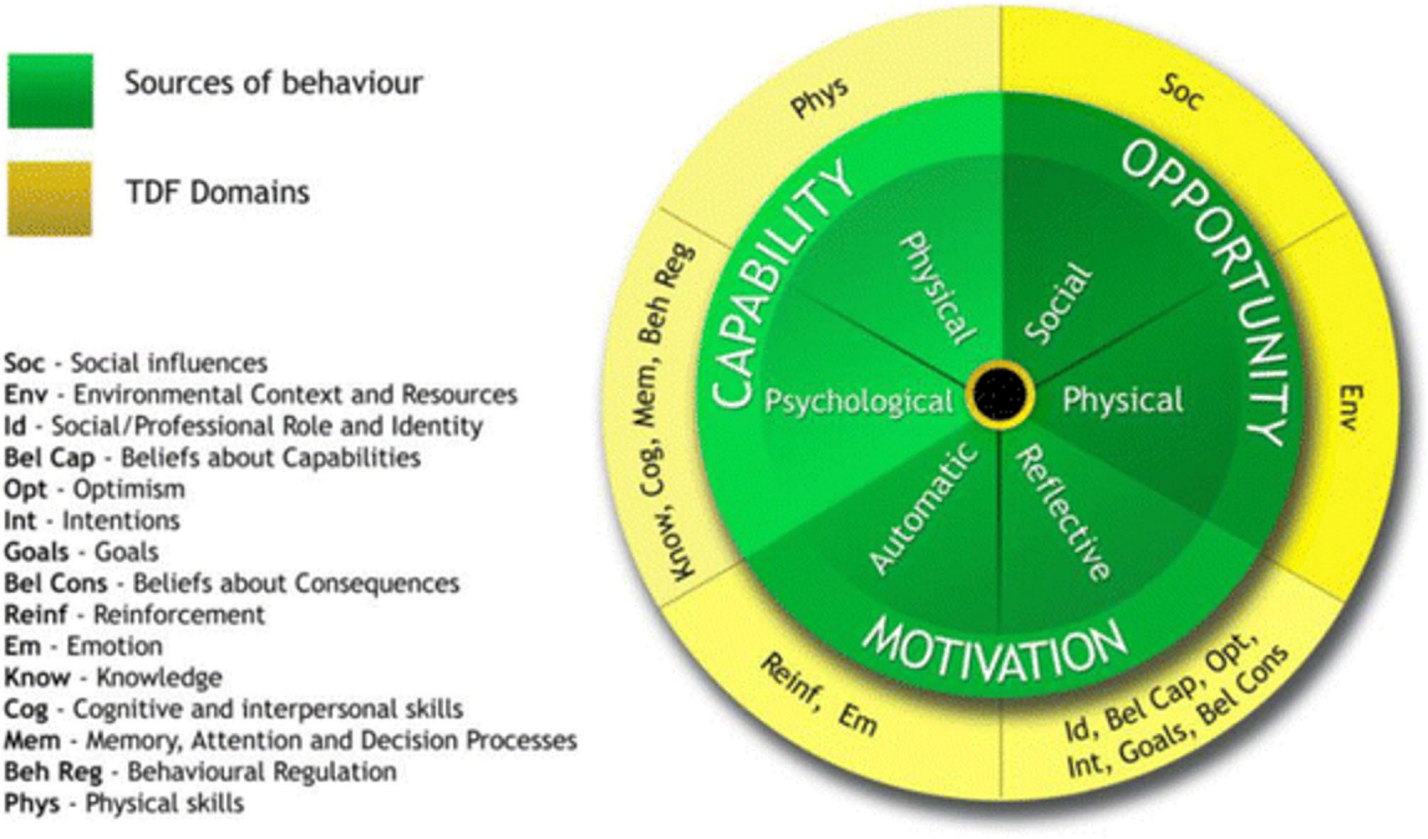
– TDF determinant links to COM-B components [29]

The BCW has been utilised within primary care and audiology services to help identify barriers and facilitators to opportunistic behaviour change interventions, and how these barriers may be overcome in future interventions [32–34]. The Capabilities, Opportunities and Motivations model of Behaviour change (COM-B) at the core of the BCW was used to inform the main interview topic guides (see supplement 1.), and explore barriers and facilitators associated with communication between the two sectors [29]. The COM-B model was not only chosen because it lies at the heart of the BCW, but because it helps overcome limitations of other theories (e.g., Health Belief Model; Taylor et al., 2006 [35]), such as helping specify intervention content, a key feature of this study. The COM-B is made up of six components that drive behaviour: physical (e.g., skills) and psychological (e.g., knowledge) capability, physical (e.g., resources) and social (e.g., social cues) opportunity, and automatic (e.g., habits) and reflective (e.g., desire) motivation. The COM-B model has previously developed topic guides to explore barriers and facilitators within primary care [34], as well as other health behaviour change contexts [36, 37]. Use of the COM-B allowed us to broadly explore barriers and facilitators, as well as link emergent themes to the 14 domains of the Theoretical Domains Framework (TDF) (e.g., knowledge, social influences, beliefs about capabilities) for a more precise analysis of barriers and facilitators that address the study aims, thus providing behavioural determinants to target for future interventions [29, 38, 39] (see Figure 1).

Further application of the BCW [29] and TDF guidelines [38] allows theoretical domains to be translated into Behaviour Change Techniques (BCTs) and intervention functions that could potentially form the basis of future complex interventions. BCTs are the observable and active components of interventions (e.g., prompts and cues), while the intervention functions are the categories by which an intervention can operate (e.g., environmental restructuring). We have provided examples of both BCTs and intervention functions that have been informed by the emergent themes and TDF domains (see Tables 1 and 2). An exemplar intervention is highlighted in the supplement material 2 guided by the BCW worksheets [29].

**Table 1:** Barriers and facilitators to communication between adult hearing aid services and general practice: general practice perspective

COM Component	TDF Domain	Themes	Explanation	Exemplar quotes	Intervention Functions	BCTs	One Exemplar Intervention
Psychological Capability	Knowledge *(An awareness of the existence of something)*	Sufficient knowledge	Staff have the procedural knowledge and competence to review and action audiology documentation were appropriate.	*“From audiology services we can get clinic letters saying that they have had the hearing test*,* or going to have an implant*,* be referred to ENT*,* x-rays or ultrasound. Large range of stuff.”*	No action	No action	No action
	Memory, attention and decision processes *(The ability to retain information*,* focus selectively on aspects of* *the environment and choose between two or more alternatives)*	•Occasional tiredness and feeling mentally drained* •Adequate stamina to complete tasks •Take a break and/or do something else.	Mental fatigue discussed. However, this does not seem to be a major concern to staff as they have adequate stamina to complete their work. Furthermore, staff take breaks and/or do other tasks in order to avoid cognitive overload.	*“Yeah*,* you know I think towards the end of the day you know that’s enough and back tomorrow.”* *“I have enough stamina.”* *“Walk away from the screen*,* go to reception*,* have a chat with a colleague*,* and then go back and crack on with it.”*	•Environmental restructuring	ϖ Adding objects to the environment ϖ Prompts and cues	Deploy positive messages within the general practice environment encouraging all staff to take regular breaks (Intervention function: environmental restructuring; BCTs: adding objects to the environment, prompts and cues) beyond their usual contractual arrangements, helping to avoid fatigue and cognitive overload.
Physical opportunity	Environmental context and resources *(Any circumstance of a person’s situation or environment that discourages or encourages the development of skills and abilities*,* independence*,* social* *competence and adaptive* *behaviour)*	•Multiple agencies can make life harder* •Sufficient information received from audiology •Sufficient time and resources available •Lack of coding on letters from audiology*	Working with multiple providers (e.g., NHS, Specsavers) can add confusion (e.g., pathways can differ, re-referred impacted wax). Administration staff feel that they receive enough information from audiology services in order to do their job effectively. Staff feel that they have enough time and resources (e.g., computers) to complete their tasks, including electronic health records (e.g., Emis, Docman). Staff would appreciate a specific code to be present on patient letters from adult hearing aid services, or for the letter to be remotely coded and uploaded, saving resources. This would also free up time and resources for further actions to be completed (e.g., further coding, e-outcomes)..	*“One of the things with audiology is where to refer people. We’ve had people saying they want to be referred to Specsavers for a hearing test. So I guess that’s probably one of the areas that is a bit more difficult. It’s quite hard to know where people have been seen before”* *“I think the audiology one’s have the appropriate amount of information”* *“We’re given enough admin time.”* *“We’ve got computers that are absolutely fine to do everything we need to do.”* *“In an ideal world information could be coded remotely”.* *“Doing my job*,* it’s difficult*,* as clear information about any follow-up they would like would help me in my job*,* personally.”*	•Environmental restructuring •Training	ϖ Adding objects to the environment ϖ Prompts and cues ϖ Demonstration of the behaviour ϖ Instruction on how to perform a behaviour ϖ Behavioural practice/rehearsal ϖ Habit formation	General practice to supply adult hearing aid services with pre-defined codes to add to their templates/letters (e.g., hearing aids fitted), to help with coding and further actions (e.g., e-outcomes). General practice staff would then be prompted to add the new codes to the patient record. (Intervention functions: environmental restructuring and training. BCTs: adding objects to the environment, prompts and cues, demonstration of the behaviour, instruction on how to perform behaviour, behavioural practice/rehearsal, habit formation).
Social opportunity	Social influences *(Those interpersonal processes that can cause individuals to change their thoughts*,* feelings*,* or behaviours)*	Collective support network between staff members	There is a team ethos within general practice. Admin staff happily seek help from one another and the general practitioner’s.	*“I can ask any GP any time and they come and happily help me. Colleagues*,* we are actually each other’s support.”*	No action	No action	No action
Reflective motivation	Social/Professional role and identity *(A coherent set of behaviours and displayed personal qualities of an individual in a social or* *work setting)*	Professionalism and patient centred	Staff are motivated by a professional identify of wanting to do their tasks to the best of their ability, as the overarching aim is to make sure information is correct for the patient.	*“If you’re going to do it*,* do it properly.”*	No action	No action	No action
Automatic motivation	Reinforcement *(Increasing the probability of a response by arranging a dependent relationship*,* or contingency*,* between the response and a given stimulus)*	Autopilot/habit	Staff are able to process adult hearing aid services documents without thinking, as their work has become an unconscious habit	*“But still*,* because I am doing it repetitively*,* those letters*,* it comes easy to me to find what I am looking for*,* in the letter.”*	No action	No action	No action

* Barriers. TDF definitions taken from Atkins et al. [39].

**Table 2:** Barriers and facilitators to communication between adult hearing aid services and general practice: adult hearing aid service perspective

**COM Component**	**TDF Domain**	**Themes**	**Explanation**	**Exemplar quotes**	**Intervention Functions**	**BCTs**	Exemplar Intervention
Psychological Capability	Knowledge *(An awareness of the existence of something)*	•Knowledge that GP services are stretched* •Not known if GPs read letters* •Knowledge of what GPs want in letters •Lack of information in referrals*	Adult hearing aid service staff are aware of the pressure on general practice, and understand that this may be impacting communication. Currently no understanding or knowledge if letters from audiologists are read by general practitioners. Audiologists would generally like to know how much information general practitioners want in letters, making them more relevant. Occasionally, it would be preferable to have more information in referrals to help with the running of clinics (e.g., interpreters required, transport issues, and any disabilities).	*“They are probably under a lot of pressure so they can’t write us a very detailed letter”* *“No, like doctors letters you sometimes wonder whether they read them. You know, whether it’s just filed, or they read the results?”* *“I mean, what would make it easier, I suppose knowing what information they want”* *“Often we get very small, I mean some referrals we get will be “please can you see this patient for a hearing test”. That is it.”*	•Education	v Information about health consequences v Information about social and environmental consequences v Feedback on behaviour v Feedback on outcomes of behaviour v Prompts and cues	Electronic notification from primary care sent to adult hearing aid services confirming that their letters have been read and actioned by the general practitioner (Intervention function: education; BCT: feedback on behaviour, feedback on outcomes of behaviour). This would primary be an intervention targeted towards general practice.
	Memory, attention and decision processes *(The ability to retain information, focus selectively on aspects of* *the environment and choose between two or more alternatives)*	•Mental fatigue, tiredness, burnout* •Weight of tasks - internal psychological pressures*	Stamina appears to have been affected due to increases in workload, staff shortages, and the effects of SARS-CoV-2Staff appear to be on edge of burnout due to demands. Both audiologists and administration staff discussed feeling the pressure of the job/number of tasks affecting their mental processes to complete clerical work. Administration staff appear to be most affected.	*“Everything is too much, everyone is snowed under, it’s too much”* *“Yeah, it’s shocking. I feel as though my brain has reached saturation point. Somebody I could’ve been talking to on the phone ten minutes ago, gone out of my head completely.” * *“Yeah, there is quite a weight of tasks that you have to do and depending on how that is at any one time, probably does have a bit of a bearing on the efficiency of, of getting some of those letters out.”*	•Environmental restructuring	v Adding objects to the environment v Prompts and cues	Similar to general practice, deploy positive messages within the audiology environment encouraging all staff to take regular breaks (Intervention function: environmental restructuring; BCTs: adding objects to the environment, prompts and cues) beyond their usual contractual arrangements, helping to avoid fatigue and cognitive overload.
Physical opportunity	Environmental context and resources *(Any circumstance of a person’s situation or environment that discourages or encourages the development of skills and abilities, independence, social competence and adaptive behaviour)*	•Limited resources, time constraints, and staff shortages* •Misuse or non-use of e-referrals systems* •Use of templates streamlines letters •Acknowledgement of letters	Administration feel that some resources are limited (e.g., direct phone lines/emails to GP services), including lack of time to complete tasks. Staffing is also affecting performances. Administration staff discussed that the current e-referral systems are misused, or not used at all. Meaning duplication, or slower workflow. Audiologists find templates to be useful tools. Staff believe some form of acknowledgement from GP services with reference to previous correspondence would be beneficial in certain situations.	*“Not enough staff”* *“They have to go through the GP so that can be a nightmare for them trying to get through”* *“We’re still getting referrals from GPs and we’re having to write back and say you need to book this through e-referral”* *“We've kind of created these templates, you know, input into that, they're quite modifiable as it stands at the moment.”* *“It would be good to get an acknowledgement from the GP if we've asked the GP to say, refer them on somewhere for an ENT service locally”*	•Training	v Demonstration of the behaviour v Instruction on how to perform a behaviour v Behavioural practice/rehearsal v Habit formation	Retraining general practice on the use of and importance of using e-referral systems correctly, which would also help to reduce burden on limited resources (Intervention function: training; BCTs: demonstration of the behaviour, instruction on how to perform behaviour, behavioural practice/rehearsal, habit formation). This would primary be an intervention targeted towards general practice.
Social opportunity	Social influences *(Those interpersonal processes that can cause individuals to change their thoughts, feelings, or behaviours)*	Group identity	Staff generally feel part of a team and supportive to one another.	*“In this department, we tend to say, what do you think about that? You know, we do swap information, and we do talk to each other about things”*	No action	No action	No action
Reflective motivation	Beliefs about consequences *(Acceptance of the truth, reality, or validity about outcomes of a behaviour in a given situation)*	•Two-way communication can be difficult* •Lack of feedback from GP services*	There are difficulties in communication between the two services, partly due to barriers associated with capabilities (e.g., stretched services) and opportunities (e.g., direct line of contact). However, the outcome of this is that it affects the planning and desire for future contact, including feedback to improve services.	*“We’re not able to have an open discussion with the GP about why that initial referral has gone wrong somewhere. We’re just left having our discussions here, and there’s no-one in between in liaison, so I thinks that’s a big shortcoming really”* *“So the last time we did a questionnaire to GP’s some years ago, and of all the GP’s I contacted, we received two replies”*	•Persuasion	v Information about social and environmental consequences v Credible source v Goal setting (behaviour) v Goal setting (outcome) v Feedback on behaviour v Feedback on outcomes of behaviour	General practice and adult hearing aid services to negotiate new approaches to communication via a third party, with a view to improving current outcome expectancies (Intervention function: persuasion; BCTs: information about social and environmental consequences, credible source, and goal setting (behaviour)). This would be a dual intervention involving both services.
	Social/Professional role and identity *(A coherent set of behaviours and displayed personal qualities of an individual in a social or* *work setting)*	Professionally motivated	It is clear that members of the adult hearing aid services are professionally motivated to do their job, including communication with primary care.	*“I know it needs doing, I just kind of get on with it. It’s part of my job.” *	No action	No action	No action

* Barriers. TDF definitions taken from Atkins et al. [39].

### Analysis

Two data sets: (1) interviews with general practice staff, and (2) interviews with hearing aid service staff; were analysed separately,. Results can be found in Tables 1 and 2.

Thematic analysis according to the six stages outlined by Braun and Clarke [40] was conducted by the first author (SK), with a full secondary analysis performed by another member of the team (MTL). MTL used NVivo to carry out analysis, while SK used pen and paper. As stated by Braun and Clarke [40] in their guidelines for thematic analysis, there was no specific quantitative threshold for what constitutes a theme, rather the value of themes relied on the researcher’s judgement and interpretation. Two levels of analysis were performed, (1) an inductive approach was used to generate the initial themes, and (2) a deductive approach was then used to map themes to relevant domains of the TDF, guided by Atkins et al. [39], and similar to that noted in Keyworth et al. [34] and Loughran et al. [37]. Each domain of the TDF can be categorised within the six components of the COM-B, as described by Michie el al. [29]. Agreement on final themes and TDF domains was reached through a series of meetings between the authors SK, MTL and CJA (see Tables 1 and 2). The identification of the precise behaviour determinants of the TDF aids the implementation of evidence-based interventions [29].

To highlight how the findings could be used to inform the design of future interventions, relevant intervention functions and BCTs were mapped to the findings of each TDF domain [29, 38] (see Tables 1 and 2). This step was strengthened by the involvement of another member of the research team (CJA).

## Results

### Information general practice require from adult hearing aid services

General practice felt that they already received sufficient information from adult hearing aid services (e.g., provision of hearing aids, onward Ear Nose and Throat [ENT] referral if required) to perform the wanted behaviour, *“Enough to code them yes” (P21)*, but that they could perhaps be refined further (see ‘*environmental context and resources*’ below). When general practice refer to the word ‘code’ this is in relation to the action of accurately recording information on their IT systems, in this instance referral information received from secondary care adult hearing aid services. There were suggestions of possible additional information for clarification purposes, such as, if general practitioner action was needed, and whether the person had a hearing loss in one or both ears.

### Information adult hearing aid services can provide to general practice

Audiologists described sending a range of information to general practice, including a summary letter of the appointment, hearing tests (audiograms), hearing aid provision (one or two), and more in-depth letters if the patient needed an onward referral; however, in contrast to what we now know from general practice staff, audiologists were unsure if this is an appropriate amount of information, *“They’re quite comprehensive actually. We do send them a lot of information. And you do wonder sometimes whether they need all of that. I mean we do it anyway”* (P09) (also see ‘*knowledge’* below). They suggested they are willing to send further information if it is required, but that this has rarely been requested in the past.

### Barriers and facilitators

Results are presented with respect to TDF domains, with descriptions of the explanatory themes for participants from general practice and adult hearing aid services compared and contrasted. The key findings presented in Tables 1 and 2 are mapped to relevant BCTs to highlight how the data could be translated to potential intervention implementation for both services.

Seven theoretical domains were identified as being able to describe the barriers and facilitators to recording and communicating hearing health care information between both services. Four domains are described in detail below (knowledge; memory, attention and decision processes; environmental context and resources; and beliefs about consequences) as these were deemed to warrant the coding of BCTs for exemplar interventions.

The remaining three domains (social influences; social/professional role and identity; reinforcement) were identified as not currently requiring attention due to only highlighting facilitators. Both sets of staff appear to have strong group identities/support networks (e.g., social influences), as well as being professionally motivated to do their jobs to the best of their abilities (e.g., social/professional role and identity). General practice staff, particularly those coding clinical outcomes have built up strong habits through repetition (e.g., reinforcement).

### Knowledge (psychological capability)

General practice staff described having sufficient knowledge, especially procedural knowledge. They appear to be competent reviewing and actioning adult hearing aid service documentation where appropriate. For example, “*From audiology services we can get clinic letters saying that they have had the hearing test*,* or going to have an implant*,* be referred to ENT*,* x-rays or ultrasound. Large range of stuff.*” *(P18).* There were no concerns about a lack of information from adult hearing aid services. If anything, they felt at times that there was too much technical or clinical information in the letters that they received. On the other hand, audiologists do not have any way of knowing whether general practitioners read their letters, “*No*,* like doctors letters you sometimes wonder whether they read them. You know*,* whether it’s just filed*,* or they read the results?*” (P07), or knowledge of the level of information general practitioners require from them, “*I mean*,* what would make it easier*,* I suppose knowing what information they want*” (P01), which would make their letters more relevant and potentially help address other barriers (see ‘*memory*,* attention and decision processes*’). It would appear that improved communication between the two services could help resolve some of these barriers.

Lack of practical information in referrals from general practice, such as ‘interpreter or transport required’ increased workload and time pressures for adult hearing aid service staff. More accurate information in referrals would help with the running of clinics (e.g., interpreters required, transport issues, and any disabilities), “*They are probably under a lot of pressure so they can’t write us a very detailed letter*” (P13) and “*Often we get very small*,* I mean some referrals we get will be “please can you see this patient for a hearing test”. That is it*” (P03). However, adult hearing aid service staff indicated knowledge and understanding that general practice services are stretched, which could be having an impact.

### Memory, attention and decision processes (psychological capability)

Mental fatigue, tiredness, and burnout were all discussed by adult hearing aid service staff in relation to the weight of tasks and internal psychological pressures. Stamina associated with cognitive overload appears to have occurred due to increases in workload, staff shortages, and the effects of SARS-CoV-2 on departments and well-being (e.g., due to the detrimental impact of how the pandemic was handled through various actions, such as, redeployment and adverse working conditions [WHO, 2025]). Both audiologists and administration staff discussed feeling the pressure of the job/number of tasks affecting their mental processes to complete clerical work, with some appearing to be on the edge of burnout, particularly administration staff, with one person stating *“Yeah*,* it’s shocking. I feel as though my brain has reached saturation point. Somebody I could’ve been talking to on the phone ten minutes ago*,* gone out of my head completely.”* and another stating that (P06) “*Everything is too much*,* everyone is snowed under*,* it’s too much*” (P06). General practice staff discussed experiencing occasional tiredness and feeling drained, and in order to help with this some have deployed their own coping mechanisms, such as taking regular breaks or switching to a different task to avoid cognitive overload, “*Walk away from the screen*,* go to reception*,* have a chat with a colleague*,* and then go back and crack on with it*” (P19). This is a tactic that could perhaps be deployed within adult hearing aid services if they have the capacity to do so. However, this does not hide the fact that many healthcare workers are overstretched with workload across various sectors, and in the need of methods of relieving the pressures of the job to avoid burnout.

### Environmental context and resources (physical opportunity)

General practice staff discussed receiving sufficient information from adult hearing aid services to do their jobs effectively “*I think the audiology ones have the appropriate amount of information*” (P25). However, a lack of physical coding on letters from adult hearing aid services was discussed in relation to increasing workload as staff were having to read the entire letter to then code it, which in turn gets checked by general practitioners. Staff would appreciate a specific code to be present on patient letters from adult hearing aid services, or for the letter to be remotely coded and uploaded, saving resources, “*In an ideal world information could be coded remotely*” (P26). They found that the lack of a code made it difficult to do some of their additional work (e.g., to action letters: further coding, e-outcomes) “*doing my job*,* it’s difficult*,* as clear information about any follow-up they would like would help me in my job*,* personally*” (P24). Audiologists discussed using pre-existing templates as useful tools to help with their workflow, “*We’ve kind of created these templates*,* you know*,* input into that*,* they’re quite modifiable as it stands at the moment*” (P01). They find these tools useful for time saving and creating more standardised responses. With templates already being used and accepted by adult hearing aid services, they could represent one way of addressing the coding issue raised by general practice without overly impacting workflow.

Time (e.g., administration staff feeling that they lack time to complete tasks) and resources (e.g., computers and use of electronic health records [EMIS, Docman]) to complete allotted tasks does not appear to be a concern for general practice staff. However, adult hearing aid service administration felt that their resources are at times limited (e.g., direct phone lines/emails to general practice), “*They have to go through the GP so that can be a nightmare for them trying to get through*” (P11). This could result in adult hearing aid service staff waiting in the queue for more than an hour to speak to the general practice about a patient. Misuse or non-use of the e-referrals systems was also reported, resulting in duplication, or slower workflow for adult hearing aid service administration, “*We’re still getting referrals from GPs*,* and we’re having to write back and say you need to book this through e-referral*” (P06). Unnecessary delays such as these are impacted further with staff shortages affecting overall performance, “*Not enough staff*” (P06).

Furthermore, adult hearing aid service staff believe some form of acknowledgement from general practice services with reference to previous correspondence would be beneficial in certain situations (also see ‘*knowledge*’), “*It would be good to get an acknowledgement from the GP if we’ve asked the GP to say*,* refer them on somewhere for an ENT service locally*” (P01).

General practice staff discussed the impact of receiving information from multiple agencies (e.g., NHS, Specsavers) and how it can add confusion and delays (e.g., pathways can differ, or re-referred impacted wax), “*One of the things with audiology is where to refer people. We’ve had people saying they want to be referred to Specsavers for a hearing test. So I guess that’s probably one of the areas that is a bit more difficult. It’s quite hard to know where people have been seen before*” (P25).

### Beliefs about consequences (reflective motivation)

Adult hearing aid service staff reported that they have encountered problems when trying to communicate with general practice, including receiving a lack of feedback on survey data sent out by management *“So the last time we did a questionnaire to GPs some years ago*,* and of all the GPs I contacted*,* we received two replies”*. As a consequence of adult hearing aid services having these difficulties trying to communicate with general practice the outcome expectancy, or anticipated consequence, is reduced motivation to make contact, *“We’re not able to have an open discussion with the GP about why that initial referral has gone wrong somewhere. We’re just left having our discussions here*,* and there’s no-one in between in liaison*,* so I think that’s a big shortcoming really” (P13)*.

### Exemplar interventions using BCTs

Exemplar interventions for both general practice and adult hearing aid services are presented in Tables 1 and 2 alongside mapped intervention functions and BCTs, in accordance with the BCW [29].

Four of the nine intervention functions from Michie et al. [28, 29] were mapped to the four key TDF domains described above: education, environmental restructuring, training, and persuasion. Nine of the 16 BCT clusters were mapped to intervention functions, domains and explanatory themes: goals and planning, feedback and monitoring, shaping knowledge, natural consequences, comparison of behaviour, associations, repetition and substitution, comparison of outcomes, and antecedents. Across these nine clusters, 13 individual BCTs were identified. If designers of interventions planned to target environmental context and resources, to address the lack of codes on letters impacting on general practice resources, they could: (1) supply a list of standardised codes on templates to adult hearing aid services to prompt use (intervention function: environmental restructuring; BCTs: adding objects to the environment, prompts and cues), and (2) provide adult hearing aid service staff with training to use these correctly, thus becoming a habit through repetition (intervention function: training; BCTs: demonstration of the behaviour, instruction on how to perform behaviour, behavioural practice/rehearsal, habit formation). The full workings of how this intervention was formulated using the worksheets from the BCW can be found in supplement 2.

## Discussion

In order to determine potential determinants of behaviour to target for interventions to improve collaboration between services, we compared two sets of healthcare professionals, general practice and adult hearing aid services. The hearing pathway is broken into seven stages [17, 18], with each stage representing an opportunity for improved efficiency and cost-effectiveness of hearing aid use within the NHS, and we identified stage four, the hearing assessment referral, as a suitable stage to investigate. Topic guides were informed by the COM-B model [28, 29] and emergent themes were mapped to the 14 determinants of the TDF [38]. Four key determinants (*knowledge; memory*,* attention and decision processes; environmental context and resources; belief about consequences*) were identified to assist with communication and the recording of referral information between these two services that could form the basis of future interventions to improve communication and collaboration.

We now know that general practice receive sufficient information (TDF: *knowledge*) on whether patients receive hearings aids, and, if anything, they perhaps receive more information than they require for their coding purposes. However, adult hearing aid services are not aware of what level of information general practice actually need. This is perhaps why too much detail is added to communications, resulting in additional time and resources being used to write and then decipher letters, unnecessarily. Time saving is one of the potential benefits of improved communication and collaboration outlined by the NHS Confederation [1].

One solution proposed by general practice staff is to streamline or customise letters sent by adult hearing aid services so that they already include the necessary codes associated with hearing aid distribution (TDF: *environmental context and resources*). This would mean that letters could be processed quicker, freeing up resources, and making the content more consistent. This is an approach that has potential to work for both sectors, as audiologists stated that they currently use templates as it improves workflow. Streamlining this part of the referral process between the two services has the potential to improve both communication and the recording of information, and a method that could be assessed and deployed across several primary and secondary care services to improve collaboration. Sharing information was one of the key areas outlined to the NHS Confederation by healthcare professional to target for improvements [1]. Deployment across multiple primary care centres would not only make the data more consistent, but accurate, timely, efficient, valid, and complete - improving the quality for audit and research purposes [2]. However, collaboration is complex, and would require organisational reshaping to help achieve change [6].

Other key findings include administration staff within adult hearing aid services feeling fatigued/tired/burnt out (TDF: *memory*,* attention and decision processes*), more so since the SARS-CoV-2 pandemic. In order to help combat some of these issues general practice administration staff highlighted that they took regular breaks from their computer outside of those normally scheduled to help relieve pressure (e.g., walk away from desk; talk to a colleague). Frequent breaks from computer-based activities has improved productivity and well-being [41], so this is potentially a tactic that could be deployed across secondary care within the NHS, if possible. However, this is only perhaps one small step to help avoid burnout and improve working conditions. Further steps like these may help retain staff and maintain high levels of care. Adult hearing aid service staff also discussed problems communicating with general practice, including non-returned survey feedback to try and improve services, which has reduced motivation to engage again (TDF: *belief about consequences*). Staff are fully aware of the pressures general practice are currently under and so have a level of understanding for their colleagues. Approachability and availability of primary care staff is a key fundamental to good communication [42]; however, perhaps recent increases in workloads are making this less feasible [43].

Working through the BCW worksheets [29], the behaviour “improved coding and recording of the supply of hearing aids” was identified as the most appropriate target behaviour to address as a worked example, involving both services (see Supplement 2). This example targets the TDF domain ‘environmental context and resources’ as the determinant to try and bring about change. It deploys the intervention functions ‘environmental restructuring’ and ‘training’ via a new adult hearing aid service clinical template with streamlined codes for hearing aids, prompts for general practice staff to add these codes to patient records, and training required for both groups of staff members. The BCTs associated with this two-way intervention included ‘adding objects to the environment’, ‘prompts and cues’, ‘demonstration of the behaviour’, ‘instruction on how to perform behaviour’, ‘behavioural practice/rehearsal’, and ‘habit formation’. This worked example has the potential to improve workflow and free up time and resources to complete other tasks for both services in line with improved collaboration outlined by the NHS Confederation [1]. Full workings and how the proposed intervention would work can be found in the supplement 2.

A preliminary study based on an adaptation of the exemplar intervention outlined above was deployed in a sample of two North West general practices. This confirmed that a new template with coding scheme can work, but implementation within practice was low, suggesting the need for refinement (e.g., deploying a further behaviour change intervention for audiologists) and evaluation. Templates and coding structures such as these may benefit from incorporation into electronic patient record systems, if applicable to the healthcare service in question.

Although this study has highlighted new findings in terms of communication between general practice and adult hearing aid services (e.g., highlighting four key determinants that could form the basis of future interventions), it is limited in perspectives taken from only two NHS services. However, in line with previous studies investigation primary care and secondary care services, there is an ongoing need for more open lines of communication and improved record sharing [1, 5]. The study has limited external validity in that the sample of departments utilised was from only one region of England, which may limit the barriers found as there may be regional differences in workflow. The exemplar intervention targeted one of four determinants and it may be worth considering interventions based on the other determinants. A key strength of this study is that it comprehensively addresses the communication and recording of referral information between general practice and adult hearing aid services in the North West of England.

## Conclusions

For the first time we identified targets for future interventions to improve communication and record sharing between general practice (i.e. primary care) and adult hearing aid services (i.e. secondary care) using the 14 domains of the TDF. Interventions should address: k*nowledge*,* environmental context and resources*, *memory*,* attention and decision processes* and *beliefs about consequences.* Future research should prioritise testing the feasibility of interventions developed using behaviour change theory, such as the exemplar outlined within this research study.

## Supplementary Information

Supplementary Material 1

Supplementary Material 2

## Data Availability

The datasets used and/or analysed during the current study are available from the corresponding author on reasonable request.

## Abbreviations

BCT: Behaviour Change Technique
BCW: Behaviour Change Wheel
COM-B: Capabilities, Opportunities and Motivations model of Behaviour
EMIS: Egton Medical Information Services
ENT: Ear Nose and Throat
GP: General Practitioner
IRAS: Integrated Research Application System
TDF: Theoretical Domains Framework
NHS: National Health Service
QoL: Quality of life
