# Supplementary material for: Identifying targets for interventions to improve communication between primary and secondary care: a qualitative study of referrals to adult NHS hearing aid services
Source: Arch Public Health. 2025 Nov 26;83:284. doi: 10.1186/s13690-025-01756-4 (PMC12659565; doi:10.1186/s13690-025-01756-4)
Supplement: Supplementary file 1 — Supplementary Material 1 [file 13690_2025_1756_MOESM1_ESM.docx]

**Schedule for Audiology Services**

- Please tell us about your current job role, experience and qualifications
- Of the patient information you send in a typical working week, what percentage is to GP practices? Where else do you send such information?
- What kinds of information do you send to GP practices?
- Do you receive any feedback from GP practices on the information you send them?
- Could you send GP Practices more information, if asked?

**Do you feel you that you have the PHYSICAL opportunity to send accurate information to GP practices?**

**What is PHYSICAL opportunity?**

The environment provides the opportunity to engage in the activity concerned.

(e.g., sufficient time, the necessary materials, reminders)

Probes: environmental context and resources

Comments?

**Do you feel you that you have the SOCIAL opportunity to send accurate information to GP practices?**

**What is SOCIAL opportunity?**

Interpersonal influences, social cues and cultural norms provide the opportunity to engage in the activity concerned

(e.g., other people engaging in the behaviour, support from colleagues)

Probes: social influences

Comments?

**Do you feel motivated to send accurate information to GP practices?**

**What is motivation?**

Conscious planning and evaluations (beliefs about what is good and bad)

(e.g., I have the desire to, I feel the need to)

Probes: social/professional identity, beliefs about capabilities, optimism, intentions, goals, beliefs about consequences

Comments?

**Is sending accurate information to GP practices something you do automatically?**

**Automatic motivation** involves doing something without thinking or having to consciously remember

(e.g. ‘is something I do before I realise I’m doing it’)

Probes: reinforcement, emotions

Comments?

**Do you feel PHYSICALLY able to send accurate information to GP practices?**

**What is physical capability?**

Having the physical skill, strength or stamina to engage in the activity concerned.

(e.g. I have sufficient physical stamina, I can overcome disability, I have sufficient physical skills)

Probes: physical skills

Comments?

**Do you feel you that you are PSYCHOLOGICALLY able to send accurate information to GP practices?**

**What is psychological capability?**

Knowledge and/or psychological skills, strength or stamina to engage in the necessary thought processes for the activity concerned.

(e.g. having the knowledge, cognitive and interpersonal skills, having the ability to engage in appropriate memory, attention and decision making processes).

Probes: knowledge, memory attention and decision processes, behavioural regulation, cognitive and interpersonal skills

Comments?

**Is there anything that we haven’t covered that you feel is important/relevant?**

**Schedule for GP Practices**

- Please tell us about your current job role, experience and qualifications
- Of the patient information you receive in a typical working week from services, what information do you receive? Where do you receive such information from?
- What kinds of information do you receive from audiology services?
- Do you feel you receive sufficient information from audiology services?
- What information do you feel is missed, if any, by audiology services?
- How are letters from audiology services stored and categorised?
- In terms of categorisation, can you see whether patients were referred by GPs or other sources?
- From the information you received from audiology services, what percentage do you think are stored and categorised adequately?

**Do you feel you that you have the PHYSICAL opportunity to process information accurately from audiology services?**

**What is PHYSICAL opportunity?**

The environment provides the opportunity to engage in the activity concerned.

(e.g., sufficient time, the necessary materials, reminders)

Probes: environmental context and resources

Comments?

**Do you feel you that you have the SOCIAL opportunity to process information accurately from audiology services?**

**What is SOCIAL opportunity?**

Interpersonal influences, social cues and cultural norms provide the opportunity to engage in the activity concerned

(e.g., other people engaging in the behaviour, support from colleagues)

Probes: social influences

Comments?

**Do you feel motivated to process information accurately from audiology services?**

**What is motivation?**

Conscious planning and evaluations (beliefs about what is good and bad)

(e.g., I have the desire to, I feel the need to)

Probes: social/professional identity, beliefs about capabilities, optimism, intentions, goals, beliefs about consequences

Comments?

**Is processing information accurately from audiology services something you do automatically?**

**Automatic motivation** involves doing something without thinking or having to consciously remember

(e.g. ‘is something I do before I realise I’m doing it’)

Probes: reinforcement, emotions

Comments?

**Do you feel PHYSICALLY able to process information accurately from audiology services?**

**What is physical capability?**

Having the physical skill, strength or stamina to engage in the activity concerned.

(e.g. I have sufficient physical stamina, I can overcome disability, I have sufficient physical skills)

Probes: physical skills

Comments?

**Do you feel you that you are PSYCHOLOGICALLY able to process information accurately from audiology services?**

**What is psychological capability?**

Knowledge and/or psychological skills, strength or stamina to engage in the necessary thought processes for the activity concerned.

(e.g. having the knowledge, cognitive and interpersonal skills, having the ability to engage in appropriate memory, attention and decision making processes).

Probes: knowledge, memory attention and decision processes, behavioural regulation, cognitive and interpersonal skills

Comments?

**Is there anything that we haven’t covered that you feel is important/relevant?**
