# Supplementary material for: Identifying targets for interventions to improve communication between primary and secondary care: a qualitative study of referrals to adult NHS hearing aid services
Source: Arch Public Health. 2025 Nov 26;83:284. doi: 10.1186/s13690-025-01756-4 (PMC12659565; doi:10.1186/s13690-025-01756-4)
Supplement: Supplementary file 2 — Supplementary Material 2 [file 13690_2025_1756_MOESM2_ESM.docx]

**Supplementary Materials 2 – BCW worksheets**

# Worksheet 1 – Define the problem in behavioural terms

This worksheet allows you to set out in behavioural terms the problem you are trying to solve and the individual, group or population involved.

| **What behaviour?** | Improving communication and recording of information between primary and secondary care (i.e. audiology) |
| --- | --- |
| **Where does the behaviour occur?** | General practice and audiology services |
| **Who is involved in performing the behaviour?** | General practice and audiology staff |

# Worksheet 2 – Select the target behaviour

**Task 1:** Generate a long list of candidate target behaviours that could bring about the desired outcome

| **Intervention designer response** |
| --- |
| Acknowledgment from primary care that audiology letters have been read  Reduction in staff fatigue and cognitive overload to improve workflow  Improved coding and recording of the supply of hearing aids  Improved knowledge of what information primary care would like in letters from audiology  Improved use of e-referral systems |

**Task 2:** Prioritise the behaviours by considering the following criteria:

1. How much of an impact changing the behaviour will have on desired outcome
2. How likely it is that the behaviour can be changed (when considering likelihood of change being achieved, think about the capability, opportunity and motivation to change of those performing the behaviour)
3. How likely it is that the behaviour (or group of behaviours) will have a positive or negative impact on other, related behaviours
4. How easy it will be to measure the behaviour

Different criteria will be more or less important in different situations. As a result of this prioritization exercise, you are likely to reach one of the following decisions:

1. The behaviour appears very promising as a target behaviour
2. The behaviour is quite promising as a target behaviour
3. The behaviour appears unpromising but is worth considering as a target behaviour
4. The behaviour is not acceptable as the target behaviour (it doesn't matter what it is like on the other criteria, this behaviour cannot be selected as the intervention target)

| **Potential target behaviours** | **Impact of behaviour change (unacceptable, unpromising but worth considering, promising, very promising)** | **Likelihood of changing behaviour (unacceptable, unpromising but worth considering, promising, very promising)** | **Spillover score**  **(unacceptable, unpromising but worth considering, promising, very promising)** | **Measurement score**  **(unacceptable, unpromising but worth considering, promising, very promising)** |
| --- | --- | --- | --- | --- |
| Acknowledgment from primary care that audiology letters have been read | Promising | Promising | Unpromising but worth considering | Very promising |
| Reduction in staff fatigue and cognitive overload to improve workflow | Promising | Unpromising but worth considering | Promising | Unpromising but worth considering |
| Improve the coding and recording of the supply of hearing aids | Promising | Very promising | Promising | Very promising |
| Improved knowledge of what information primary care would like in letters from audiology | Promising | Very promising | Promising | Promising |
| Improved use of e-referral systems | Promising | Promising | Promising | Promising |
| **Record selected target behaviour here:** | Improved coding and recording of the supply of hearing aids | | | |

# Worksheet 3 – Specify the target behaviour

**Task:** Describe the target behaviour according to who, needs to do what, when, where, how often and with whom

| **Target behaviour** | Improved coding and recording of the supply of hearing aids |
| --- | --- |
| ***Who* needs to perform the behaviour?** | Primary and secondary care staff |
| ***What* do they need to do differently to achieve the desired change?** | Streamlined coding system |
| ***When* do they need to do it?** | After hearing aids administered by audiology and when letters received by primary care |
| ***Where* do they need to do it?** | Letters and patient records |
| ***How often* do they need to do it?** | Each time patient receives hearing aids after a referral by GP |
| ***With whom* do they need to do it?** | Alone |

# Worksheet 4 – Identify what needs to change

**Task:** Use the COM-B model/TDFs to identify what needs to change in order for the target behaviour to occur. If a more detailed understanding of the behaviour is required, use the TDF to expand on COM-B components identified.

See Table … in main text of paper.

# Worksheet 5 – Identify intervention functions

**Task:** Use the APEASE criteria to identify appropriate intervention functions based on the behavioural diagnosis arrived at in Step 4:

- Affordability
- Practicability
- Effectiveness and cost-effectiveness
- Acceptability
- Side-effects/safety
- Equity

| **Candidate intervention functions** | **Does the intervention function meet the APEASE criteria (affordability, practicability, effectiveness/cost-effectiveness, acceptability, side-effects/safety, equity)?** |
| --- | --- |
| **Environmental restructuring –** Changing the physical or social context | **Environmental context and resources –** (Adding new template, codes, and software prompts) adding new templates and codes to audiology way of working and adding new prompts to primary care software – **APEASE met** |
| **Training –** Imparting skills | **Environmental context and resources -** (Using new template, codes, and software prompts) to train audiology staff on the new templates and coding of hearing aid distribution, and to train primary care staff on adding the codes to the patient records via the digital prompts – **APEASE met** |
| **Education** | N/A |
| **Persuasion** | N/A |
| **Incentivisation** | N/A |
| **Coercion** | N/A |
| **Restriction** | N/A |
| **Modelling** | N/A |
| **Enablement** | N/A |
| **Selected intervention functions:** | Environmental context and resources; training |

# Worksheet 6 – Identify policy categories

**Task:** Use the APEASE criteria to identify appropriate policy categories based on the intervention functions identified in Step 5:

- Affordability
- Practicability
- Effectiveness and cost-effectiveness
- Acceptability
- Side-effects/safety
- Equity

| **Intervention function** | **Policy categories** | **Does the policy category meet the APEASE criteria (affordability, practicability, effectiveness/cost-effectiveness, acceptability, side-effects/safety, equity)?** |
| --- | --- | --- |
| Environmental restructuring | Guidelines  Environmental/social planning | APEASE met – i.e. changing and/or adding templates  APEASE met – i.e. adding prompts and cues |
| Training | Guidelines | APEASE met – same as above |

# Worksheet 7 – Identify BCTs

**Task:** Use the APEASE criteria to identify appropriate BCTs based on the intervention functions identified in Step 5:

- Affordability
- Practicability
- Effectiveness and cost-effectiveness
- Acceptability
- Side-effects/safety
- Equity

| **Intervention function** | **Individual BCTs** | **Does the BCT meet the APEASE criteria (affordability, practicability, effectiveness/cost-effectiveness, acceptability, side-effects/safety, equity)?** |
| --- | --- | --- |
| **Environmental restructuring** | - **Adding objects to the environment** - **Prompts and cues** | - **APEASE met.** New templates and codes added for audiology to use - **APEASE met.** Prompts added to primary care software for coding |
| **Training** | - **Demonstration of the behaviour** - **Instruction on how to perform a behaviour** - **Behavioural practice/rehearsal** - **Habit formation** | - **APEASE met.** Demonstrate to staff how to use new templates and add codes - **APEASE met.** Advise staff on how to use new templates and add codes - **APEASE met.** Prompt staff to practice using new templates and codes - **APEASE met.** Primary care staff prompted to add codes to patient records |
|  | | |

**Task:** Based on the identified intervention functions, policy categories and BCTs, draft an intervention strategy describing how BCTs will be delivered in your context.

| **Intervention functions** | **COM-B components served by intervention functions** | **BCTs to deliver intervention functions** | **Policy categories through which BCTs can be delivered** | **Intervention strategy** |
| --- | --- | --- | --- | --- |
| **Environmental restructuring and Training** | - Psychological Capability - Physical Opportunity | 1. Adding objects to the environment 2. Prompts and cues 3. Demonstration of the behaviour 4. Instruction on how to perform a behaviour 5. Behavioural practice / rehearsal 6. Habit formation | - Guidelines - Environmental / social planning | Two part intervention:  Part One - Designing a new updated audiology template, and  Part Two – Implementing a new clinical system in primary care.  Part One: Designing a new updated audiology template which includes three new questions on the letter that goes to the GP once a new patient has been assessed and provided with new hearing aids. These three changes include: (i)  Action for GP required – Yes/No, (ii) Seen in audiology code (code to be decided locally), (iii) Hearing aid provision code (code to be decided locally).  The YES/NO “action for GP” was suggested by primary care staff to reduce time processing letters. If there is no action required by the GP, it can go straight to the coding team.  Part Two: Implementing a new clinical system in primary care included designing a clinical template that would prompt primary care staff to add the code to the patient file once they had been seen in audiology. This pop-up prompt would keep appearing until the code was added. The only way to stop the prompt is to either add a hearing aid code on the same date as the clinic attendance or to tick that procedure was not done. |

# Worksheet 8 – Identify mode of delivery

**Task**: Use the APEASE criteria to identify an appropriate mode of delivery:

- Affordability
- Practicability
- Effectiveness and cost-effectiveness
- Acceptability
- Side-effects/safety
- Equity

| **Environmental restructuring and Training**  **Mode of delivery** | | | | **Does the mode of delivery meet the APEASE criteria (affordability, practicability, effectiveness/cost-effectiveness, acceptability, side-effects/safety, equity)?** |
| --- | --- | --- | --- | --- |
| **Face-to-face** | **Individual** | | | APEASE met |
|  | **Group** | | | APEASE met |
| **Distance** | **Population-level** | **Broadcast media** | **TV** | N/A |
|  |  |  | **Radio** | N/A |
|  |  | **Digital media** | **Internet** | APEASE met |
|  |  |  | **Mobile phone app** | N/A |
|  |  | **Print media** | **Newspaper** | N/A |
|  |  |  | **Leaflet** | APEASE met |
|  |  | **Outdoor media** | **Billboard** | N/A |
|  |  |  | **Poster** | N/A |
|  | **Individual-level** | **Phone** | **Phone helpline** | N/A |
|  |  |  | **Mobile phone text** | N/A |
|  |  | **Individually accessed computer programme** | | N/A |
